# The Value of Perioperative Chemotherapy for Patients With Hepatoid Adenocarcinoma of the Stomach Undergoing Radical Gastrectomy

**DOI:** 10.3389/fonc.2021.789104

**Published:** 2022-01-10

**Authors:** Kai Zhou, Anqiang Wang, Jingtao Wei, Ke Ji, Zhongwu Li, Xin Ji, Tao Fu, Ziyu Jia, Xiaojiang Wu, Ji Zhang, Zhaode Bu

**Affiliations:** ^1^ Gastrointestinal Cancer Center, Key Laboratory of Carcinogenesis and Translational Research (Ministry of Education), Peking University Cancer Hospital and Institute, Beijing, China; ^2^ Department of Pathology, Key Laboratory of Carcinogenesis and Translational Research (Ministry of Education), Peking University Cancer Hospital and Institute, Beijing, China

**Keywords:** hepatoid adenocarcinoma of stomach, neoadjuvant chemotherapy, adjuvant chemotherapy, prognosis, propensity score matching

## Abstract

**Background:**

Hepatoid adenocarcinoma of the stomach (HAS) is a rare type of gastric cancer, but the role of perioperative chemotherapy is still poorly understood. The aim of this retrospective study was to investigate the associations between perioperative chemotherapy and prognosis of HAS.

**Method:**

We retrospectively analyzed patients with locally advanced HAS who received radical surgery in Peking University Cancer Hospital between November 2009 and October 2020. Patients were divided into neoadjuvant chemotherapy-first (NAC-first) group and surgery-first group. The relationships between perioperative chemotherapy and prognosis of HAS were analyzed using univariate, multivariate survival analyses and propensity score matching analysis (PSM).

**Results:**

A total of 100 patients were included for analysis, including 29 in the NAC-first group and 71 in the surgery-first group. The Her-2 amplification in HAS patients was 22.89% (19/83). For NAC-first group, 4 patients were diagnosed as tumor recession grade 1 (TRG1), 4 patients as TRG 2, and 19 patients as TRG 3. No significant difference in prognosis between the surgery-first group and the NAC-first group (*P*=0.108) was found using PSM analysis. In the surgery-first group, we found that the survival rate was better in group of ≥6 cycles of adjuvant chemotherapy than that of <6 cycles (*P*=0.013).

**Conclusion:**

NAC based on platinum and fluorouracil may not improve the Overall survival (OS) and Disease-free survival time (DFS) of patients with locally advanced HAS. Patients who received ≥6 cycles of adjuvant chemotherapy had better survival. Therefore, the combination treatment of radical gastrectomy and sufficient adjuvant chemotherapy is recommended for patients with locally advanced HAS.

## Introduction

Hepatoid adenocarcinoma is characterized as histologically resembling hepatocellular carcinoma (HCC) with enteroblastic differentiation (1, 2). Hepatoid adenocarcinoma has been found in many extrahepatic organs, such as the stomach, ovary, gallbladder, colon, bladder, renal pelvis, lung, duodenum and pancreas, among which the stomach is the most prevalent (3–8). During the development of the human embryo, both the stomach and liver are primitive foregut derivatives and originate from the endoderm. Some gastric cancer cells may differentiate into early embryonic hepatocytes and then form hepatoid carcinoma of the stomach (HAS) (9, 10). Bourreille et al. reported the first case in 1970, a unique entity of gastric malignant tumor producing alpha-fetoprotein (AFP) with liver metastasis (11). Kodama et al. found that gastric cancer with AFP production had a well-differentiated papillary or tubular type and medullary type, and the latter was considered as hepatocellular carcinoma (12). In 1985, Ishikura et al. eventually expressly provided the term “hepatoid carcinoma of the stomach” (HAS) (13).

HAS is a rare subtype of gastric cancer (GC) that was previously reported to account for 0.38-1.6% of GC (5, 14). HAS mostly occurs in elderly male individuals without specific clinical manifestations and imaging features (15–17). HAS is mainly located in the gastric antrum and is prone to vascular invasion and early metastasis, specifically to the lymph nodes, liver and lung (15, 18, 19). According to current research, the treatment strategy for HAS is similar to gastric adenocarcinoma (15, 18). Radical surgery and adjuvant therapy are the standard treatments for resectable HAS (15, 20, 21). However, early disease recurrence and poor patient prognosis were still observed despite radical surgery with free margins (22, 23). Drugs for gastric cancer have been used as adjuvant chemotherapy (AC) or neoadjuvant chemotherapy (NAC) for a limited number of patients with HAS (5, 23, 24), and there are no definitive specific chemotherapy regimens that are beneficial for patients with HAS. In summary, there is no unanimous conclusion on the most appropriate therapeutic strategy for HAS (16).

Theoretically, NAC can resolve micrometastatic lesions (25) and alleviate disease development, thus reducing the overall mortality rate of patients with cancer. NAC provides a valuable opportunity to evaluate the effectiveness of chemotherapy, which is one of the standard treatments for advanced gastric cancer (26). However, the true effect of NAC for gastric cancer is unknown (27). The results of some studies have suggested that NAC may lead to short-term postoperative complications, which delay the implementation of AC after surgery. If the NAC protocol is ineffective against GC, there is a risk of cancer progression during the period of NAC treatment (28). However, due to the scarcity of the literature, there is minimal information available on the role of perioperative chemotherapy for HAS. Accordingly, we conducted a single-center retrospective study to elucidate the effects of NAC and AC in patients with HAS and the prognostic factors related to HAS.

## Materials and Methods

### Enrollment of Patients

We consecutively enrolled patients with HAS who underwent curative total or partial gastrectomy with D2 lymph nodes (LNs) dissection between November 2009 and October 2020. We selected patients who were pathologically diagnosed with HAS after radical gastrectomy and with clinical stage T3/T4 or N+ disease. The exclusion criteria were as follows: (1) perioperative death, (2) R0 resection was not performed, (3) preoperative or postoperative radiotherapy, (4) clinical stage IVb, and (5) pathological stage I patients without high risk factors, which included age below 40 years old, poor differentiation and lymphovascular invasion. Patients who received preoperative chemotherapy were defined as neoadjuvant chemotherapy-first (NAC-first) group, and patients who did not receive preoperative treatment were defined as surgery-first group. Clinicopathological features were retrospectively collected and all patients were followed up. We used abdominal and pelvic computed tomography (CT) to assess the clinical stage using the American Joint Committee on Cancer/Union for International Cancer Control 8^th^ classification system. Enlarged LNs over 8 mm at their largest axis or with internal necrosis were classified as cN+. This retrospective study was performed according to the tenets of the Declaration of Helsinki and was approved by the Institutional Review Board of Peking University Cancer Hospital.

**Figure 1:**
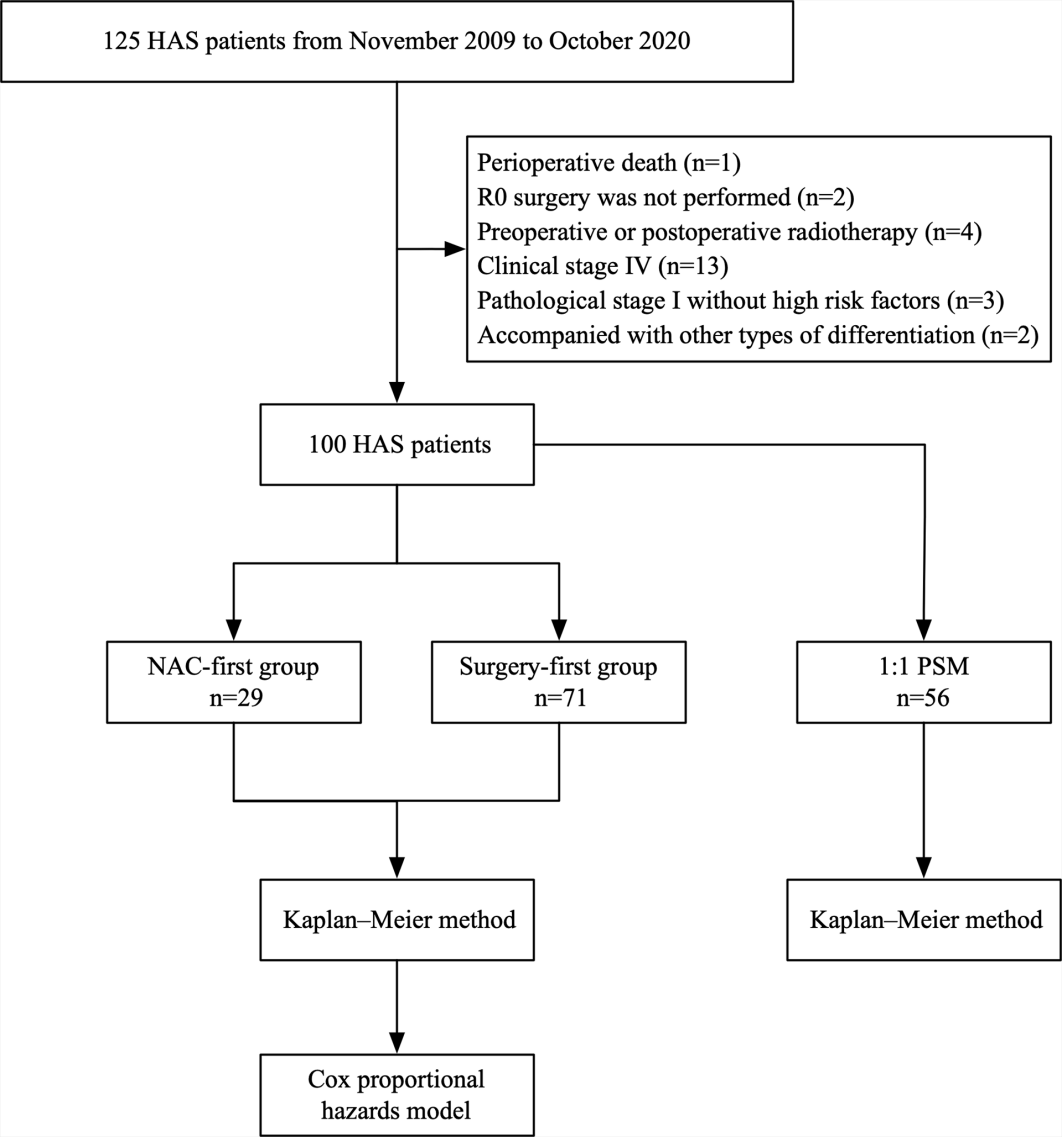
Schematic of the study design. The chart showed the selection of patients and study methods.

A total of 125 HAS patients were eligible for the study. Twenty-five patients were excluded, of whom 13 patients were diagnosed with distant metastases, 2 patients had postoperative residual lesions, 4 patients accepted perioperative radiotherapy, one patient died perioperatively and 5 patients were diagnosed with pathological stage I without high risk factors or other types of tumor differentiation. Eventually, 29 patients were included in the NAC-first group and 71 patients were included in the surgery-first group, for a total of 100 patients (**Figure 1**).

### Pathological Diagnosis and Treatment Evaluation

Pathological diagnosis was based on morphological features and immunohistochemistry, including hepatoid and/or adenocarcinoma components, by two independent pathologists (**Supplementary Figure 1**). Clinical responses to NAC were assessed based on CT scans according to the Response Assessment Criteria for Solid Tumors version 1.1 (29). The assessment of target lesions was divided into the following four categories: complete remission (CR), partial remission (PR), stable disease (SD), and progressive disease (PD). According to the NCCN guidelines for gastric cancer (2018), the pathological response was graded according to the 3-point tumor regression grading (TRG) system (30). The tumors were divided into the following four grades: grade 0 (no visible cancer cells), grade 1 (single cells or small groups of cancer cells), grade 2 (residual cancer outgrown by fibrosis) and grade 3 (significant fibrosis outgrown by cancer or no fibrosis with extensive residual cancer). We also evaluated the toxicities related to NAC by the WHO standard criteria.

### Follow-Up

The patients underwent follow-up gastroscopy, abdominal and pelvic computed tomography, chest radiography and tumor biomarkers at our hospital or local hospital 3 months after the operation and every 3 or 6 months thereafter. Overall survival time (OS) was defined as the length of time from the date of first NAC treatment or radical gastrectomy to the date of the last follow-up or the date of death from any cause. Disease-free survival time (DFS) was defined as the length of time from the date of first NAC treatment or radical gastrectomy to the date of disease recurrence, metastasis or death from any cause or the date of last follow-up. The mean follow-up time was 30.5 months, ranging from 2.4 to 102.6 months.

### Statistical Analysis

To compare the clinicopathological features of the NAC-first and surgery-first groups, SPSS 23.0 was used for statistical analysis. Independent sample *t-*tests were used for continuous variables. The chi-square test or Mann-Whitney *U* test was used for categorical variables. Propensity score matching analysis (PSM) was used to reduce the impact of possible confounding factors. The 1:1 PSM method (match tolerance 0.2) was conducted to compare the NAC-first and surgery-first groups. To estimate the long-term OS and DFS outcomes, the Kaplan–Meier method and a log-rank test were used. To evaluate the independent predictors of OS and DFS, variables with *P*<0.10 in univariate survival analyses or with clinical significance were entered into the multiple regression analysis using the Cox proportional hazards model. *P*<0.05 was considered statistically significant. GraphPad Prism 5 was used to draw the Kaplan-Meier survival curve.

## Results

### Clinicopathologic Features of Included Patients and PSM

We found that there were differences in sex, tumor location, clinical T, N, TNM stage, lymphovascular invasion, nerve invasion, PDL-1 and SALL4 expression between the NAC-first group and the surgery-first group (*P*<0.1). The positive expression rate of Her-2 (staining by immunohistochemistry 3+ or with positive fluorescence *in situ* hybridization) in HAS patients was 22.89% (19/83). In the surgery-first group, 23% (14/61) of the patients were Her-2 positive. In the NAC-first group, 22.7% (5/22) of the patients were Her-2 positive. In addition, 91.7% (88/96) of the patients had AFP-positive cells as determined by immunohistochemistry. The number of patients with stage cIII/IVa disease in the NAC-first group was substantially higher than that in the surgery-first group (100% versus 78.9%, *P*=0.01). However, lymphovascular invasion were more prevalent in the surgery-first group than in the NAC-first group (67.6% vs 44.8%, *P*=0.03) (**Table 1**). Nerve invasion levels were also similar (63.4% vs 41.4%, *P*=0.04) (**Table 1**). To reduce confounding bias, 1:1 PSM was performed, and 56 patients were ultimately included. Most clinicopathological features were not significantly different between the two groups after 1:1 PSM (**Table 1**).

**Table 1:** Baseline demographics of the study population before and after propensity score matching.

Clinicopathological features		Before PSM			After 1:1 PSM		
		Surgery-first	NAC-first	P value	Surgery-first	NAC-first	P value
		N=71	N=29		N=28	N=28	
Age (year)		61.056 ± 10.70	59.379 ± 7.50	0.44	62 ± 8.2642	59.714 ± 7.423	0.28
Gender	Female	22 (31)	3 (10.3)	0.03	3 (10.7)	2 (7.1)	1.00
	Male	49 (69)	26 (89.7)		25 (89.3)	26 (92.9)	
KPS score	80	2 (2.8)	0 (0)	0.92	–	–	0.32
	90	14 (19.7)	7 (24.1)		4 (14.3)	7 (25)	
	100	55 (77.5)	22 (75.9)		24 (85.7)	21 (75)	
Location of tumor	GEJ	15 (21.10)	13 (44.8)	0.02	9 (32.1)	13 (46.4)	0.27
	Non-GEJ	56 (78.9)	16 (55.2)		19 (67.9)	15 (53.6)	
Family history of cancer	No	56 (78.9)	22 (75.9)	0.74	22 (78.6)	21 (75)	0.75
	Yes	15 (21.1)	7 (24.1)		6 (21.4)	7 (25)	
Clinical T stage	1	2 (2.8)	0 (0)	0.01	–	–	0.17
	2	3 (4.2)	0 (0)		–	–	
	3	34 (47.9)	8 (27.6)		13 (46.4)	8 (28.6)	
	4a/b	32 (45.1)	21 (72.4)		15 (53.6)	20 (71.4)	
Clinical N stage	–	11 (15.5)	0 (0)	0.03	1 (3.6)	0 (0)	0.32
	+	60 (84.5)	29 (100)		27 (96.4)	28 (100)	
Clinical TNM stage	IIa	5 (7)	0 (0)	0.01	–	–	0.32
	IIb	10 (14.1)	0 (0)		1 (3.6)	0 (0)	
	III	55 (77.5)	28 (96.6)		27 (96.4)	28 (100)	
	IVa	1 (1.4)	1 (3.4)		–	–	
Number of lymph node dissection	<16	0 (0)	2 (6.9)	0.42	0 (0)	2 (7.1)	0.21
	16-30	30 (42.3)	12 (41.4)		9 (32.1)	11 (39.3)	
	>30	41 (57.7)	15 (51.7)		19 (67.9)	15 (53.6)	
AC	No	7 (10.3)	3 (10.7)	1.00	0 (0)	3 (11.1)	0.24
	Yes	61 (89.7)	25 (89.3)		27 (100)	24 (88.9)	
Cycles of perioperative chemotherapy	0	7 (10.3)	0 (0)	0.617			0.535
	<6	14 (20.6)	8 (28.6)		6 (22.2)	8 (29.6)	
	≥6	47 (69.1)	20 (71.4)		21 (77.8)	19 (70.4)	
Borrmann type	I	4 (7)	1 (4.2)	0.84	3 (13.6)	1 (4.3)	0.95
	II	15 (26.3)	6 (25)		4 (18.2)	6 (26.1)	
	III	36 (63.2)	17 (70.8)		14 (63.6)	16 (69.6)	
	IV	2 (3.5)	0 (0)		1 (4.5)	0 (0)	
Degree of differentiation	High/middle differentiation	43 (60.6)	17 (58.6)	0.86	19 (67.9)	16 (57.1)	0.41
	Low/undifferentiation	28 (39.4)	12 (41.4)		9 (32.1)	12 (42.9)	
Lauren type	Intestinal type	35 (49.3)	19 (65.5)	0.26	13 (46.4)	19 (67.9)	0.08
	Diffuse type	12 (16.9)	2 (6.9)		1 (3.6)	2 (7.1)	
	Mixed type	24 (33.8)	8 (27.6)		14 (50)	7 (25)	
Surgery type	Proximal gastrectomy	3 (4.2)	2 (6.9)	0.74	0 (0)	2 (7.1)	0.79
	Distal gastrectomy	37 (52.1)	13 (44.8)		14 (50)	12 (42.9)	
	Total gastrectomy	31 (43.7)	14 (48.3)		14 (50)	14 (50)	
Lymphovascular invasion	–	23 (32.4)	16 (55.2)	0.03	13 (46.4)	15 (53.6)	0.59
	+	48 (67.6)	13 (44.8)		15 (53.6)	13 (46.4)	
Nerve invasion	–	26 (36.6)	17 (58.6)	0.04	15 (53.6)	16 (57.1)	0.79
	+	45 (63.4)	12 (41.4)		13 (46.4)	12 (42.9)	
Postoperative metastasis	no	60 (84.50)	21 (72.4)	0.16	24 (85.7)	20 (71.4)	0.19
	yes	11 (15.5)	8 (27.6)		4 (14.3)	8 (28.6)	
C-met	–	8 (11.6)	2 (7.7)	0.99	3 (10.7)	2 (8)	0.79
	+	37 (53.6)	15 (57.7)		14 (50)	14 (56)	
	++	19 (27.5)	9 (34.6)		9 (32.1)	9 (36)	
	+++	5 (7.2)	0 (0)		2 (7.1)	0 (0)	
EGFR	–	2 (2.9)	1)3.8)	0.18	0 (0)	1 (4)	0.90
	+	10 (14.5)	2 (7.7)		1 (3.6)	2 (8)	
	++	38 (55.1)	12 (46.2)		18 (64.3)	12 (48)	
	+++	19 (27.5)	11 (42.3)		9 (32.1)	10 (40)	
HER-2	-/±	47 (68.1)	17 (63)	0.54	19 (67.9)	16 (61.5)	0.53
	++	8 (11.6)	5 (18.5)		3 (10.7)	5 (19.2)	
	+++	14 (20.3)	5 (18.5)		6 (21.4)	5 (19.2)	
MMR	pMMR	58 (98.3)	24 (100)	1.00	22 (95.7)	23 (100)	0.32
	dMMR	1 (1.7)	0 (0)		1 (4.3)	0 (0)	
PDL1	≤5%	39 (88.6)	9 (64.3)	0.09	19 (95)	9 (64.3)	0.02
	>5%	5 (11.4)	5 (35.7)		1 (5)	5 (35.7)	
Ki-67	<25%	3 (4.4)	0 (0)	0.23	–	–	0.79
	25-49%	6 (8.8)	1 (3.8)		1 (3.7)	1 (4)	
	50-75%	27 (39.7)	10 (38.5)		12 (44.4)	10 (40)	
	>75%	32 (47.1)	15 (57.7)		14 (51.9)	14 (56)	
SALL4	<25%	45 (68.2)	21 (91.3)	0.04	15 (53.6)	20 (90.9)	0.01
	25-49%	6 (9.1)	0 (0)		3 (10.7)	0 (0)	
	50-75%	10 (15.2)	1 (4.3)		7 (25)	1 (4.5)	
	>75%	5 (7.6)	1 (4.3)		3 (10.7)	1 (4.5)	
AFP	–	5 (7.2)	3 (11.1)	0.84	0 (0)	3 (11.5)	0.11
	+	64 (92.8)	24 (88.9)		28 (100)	23 (88.5)	
CEA (ng/ml)	0-5	44 (64.7)	15 (55.6)	0.41	17 (60.7)	14 (53.8)	0.61
	>5	24 (35.3)	12 (44.4)		11 (39.3)	12 (46.2)	
CA199 (U/ml)	0-37	58 (85.3)	25 (92.60	0.34	24 (85.7)	24 (92.3)	0.45
	>37	10 (14.7)	2 (7.4)		4 (14.3)	2 (7.7)	
CA242 (U/ml)	0-20	37 (88.1)	11 (84.6)	0.74	11 (84.6)	11 (84.6)	1.00
	>20	5 (11.9)	2 (15.4)		2 (15.4)	2 (15.4)	
CA72.4 (U/ml)	0-6.7	59 (86.8)	25 (92.6)	0.43	25 (89.3)	24 (92.3)	0.71
	>6.7	9 (13.2)	2 (7.4)		3 (10.7)	2 (7.7)	
AFP (ng/ml)	0-7	25 (48.1)	8 (40)	0.54	7 (38.9)	8 (40)	0.95
	>7	27 (51.9)	12 (60)		11 (61.1)	12 (60)	

KPS, Karnofsky Performance Status; GEJ, Gastroesophageal junction; AC, Adjuvant chemotherapy; PSM, Propensity score matching analysis; MMR, Mismatch repair.

### Regimens, Cycles, Adverse Effects and Clinical Response to NAC

In our study, 29 patients underwent NAC. Of these, 19 patients received S-1+oxaliplatin (SOX), 6 patients received oxaliplatin + capecitabine (XELOX), one received SOX+ paclitaxel + trastuzumab, one received XELOX+ trastuzumab, one received docetaxel+ cisplatin+ fluorouracil (DCF) and one received oxaliplatin+ calcium folinate+ fluorouracil (mFOLFOX). The median course of NAC was 3 cycles (1-5 cycles).

In the SOX regimen, one patient developed grade 1 gastrointestinal discomfort, and the main clinical manifestation was nausea and vomiting. One patient developed grade 1 gastrointestinal discomfort and neurotoxicity. Fourteen patients did not exhibit side effects during NAC. In the XELOX regimen, one patient experienced grade 3 gastrointestinal discomfort and grade 2 thrombocytopenia. One patient developed grade 1 neutropenia and leukopenia, grade 2 thrombocytopenia and slight numbness in the extremities. In the SOX+ paclitaxel + trastuzumab, DCF and mFOLFOX regimens, no toxicities were observed during NAC.

In the NAC-first group, a total of 6 patients achieved partial remission (PR), 20 patients achieved stable disease (SD), 1 patient had progressive disease (PD) and none achieved complete remission (CR). In the SOX regimen, pathological responses of TRG 1, 2 and 3 were observed in 2, 2 and 13 patients, respectively. In the XELOX regimen, TRG 1, 2 and 3 were observed in 0, 0, and 6 patients, respectively. Pathological responses of other chemotherapy regimens were shown in **Table 2**.

**Table 2:** Evaluation of radiological response, TRG, and main toxicity occurring of NAC.

NAC regimen		SOX (n=19)	XELOX (n=6)	SOX+ Paclitaxel + Trastuzumab (n=1)	XELOX+ Trastuzumab (n=1)	DCF (n=1)	Mfolfox (n=1)
Radiological response	CR	0 (0)	0 (0)	0 (0)	0 (0)	0 (0)	0 (0)
	PR	3 (15.8)	2 (33.3)	1 (100)	0 (0)	0 (0)	0 (0)
	SD	14 (73.7)	3 (50.0)	0 (0)	1 (100)	1 (100)	1 (100)
	PD	0 (0)	1 (16.7)	0 (0)	0 (0)	0 (0)	0 (0)
	Unknown	2 (10.5)	0 (0)	0 (0)	0 (0)	0 (0)	0 (0)
TRG	0	0 (0)	0 (0)	0 (0)	0 (0)	0 (0)	0 (0)
	1	2 (10.5)	0 (0)	1 (100)	1 (100)	0 (0)	0 (0)
	2	2 (10.5)	0 (0)	0 (0)	0 (0)	1 (100)	1 (100)
	3	13 (68.5)	6 (100)	0 (0)	0 (0)	0 (0)	0 (0)
	Unknown	2 (10.5)	0 (0)	0 (0)	0 (0)	0 (0)	0 (0)
Adverse event^a^	Gastrointestinal discomfort	2 (10.5)	1 (16.7)	0 (0)	0 (0)	0 (0)	0 (0)
	Myelosuppression	0 (0)	1 (16.6)	0 (0)	0 (0)	0 (0)	0 (0)
	No	14 (73.7)	1 (16.6)	1 (100)	0 (0)	1 (100)	1 (100)
	Unknown	3 (15.8)	3 (50.0)	0 (0)	1 (100)	0 (0)	0 (0)

a The main toxicity occurring of NAC were recorded, and the secondary side effects were not taken into account.

NAC, neoadjuvant chemotherapy; CR, complete remission; PR, partial remission; SD, stable disease; PD, progressive disease; TRG, tumor regression grade; XELOX, oxaliplatin + capecitabine; SOX, S-1+oxaliplatin; DCF, Docetaxel+ cisplatin+ fluorouracil.

### No Significant Prognostic Difference Was Associated With NAC in HAS

**Figure 2:**
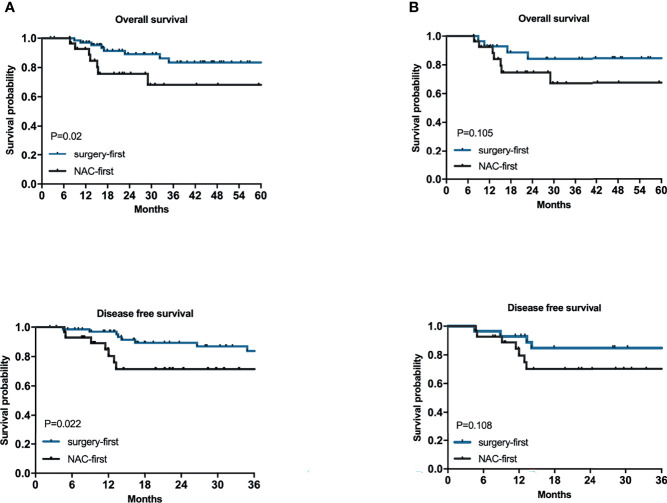
The relationships between NAC and the prognosis of HAS. Kaplan–Meier survival plots for NAC-first and surgery-first groups for 100 patients **(A)** and for after 1:1 PSM of 56 patients **(B)**. P values were calculated by the log-rank test.

The OS time of the surgery-first group was better than that of the NAC-first group (**Figure 2A**, *P*=0.02). In particular, the 1- and 3-year survival rates of the NAC-first group were 92.7% and 68.2%, respectively. The 1- and 3-year survival rates of the surgery-first group were 97% and 83.4%, respectively. And univariate survival analysis was demonstrated in **Supplementary Material**. Multivariate Cox regression analysis indicated that clinical T4 (*P*=0.015), proximal gastrectomy (*P*=0.021), lymphovascular invasion (*P*=0.030) and CA199 (*P*=0.007) were independent risk factors for poor OS outcomes in HAS patients (**Table 3**). However, no significant difference in OS times was found between the NAC-first group and the surgery-first group after PSM analysis, although the surgery-first group had a tendency toward better OS rates than the NAC-first group (**Figure 2B**, *P*=0.105).

**Table 3:** Multivariate analysis of overall survival (OS) and disease-free survival time (DFS) before propensity score matching analysis.

Clinicopathological features		OS				DFS			
		*P* value	HR	95%CI		*P* value	HR	95%CI	
Age(year)		0.091				0.104			
Location of tumor GEJ vs non-GEJ		0.164							
Clinical T stage	T1/2/3 vs T4	0.015	8.945	1.542-51.872		0.023	3.630	1.190-11.077	
Clinical N stage	N- vs N+	0.437				0.641			
Clinical TNM stage	IIa	0.943				0.411			
	IIb	0.624				0.913			
	III	0.533				0.958			
	IVa	0.763				0.441			
NAC	No vs Yes	0.115				0.265			
AC	No vs Yes	0.417				0.405			
Surgery type	Proximal gastrectomy	0.021				0.140			
	Distal gastrectomy	0.006	0.027	0.002-0.352		0.078			
	Total gastrectomy	0.034	0.068	0.006-0.813		0.051			
Number of lymph node dissection		0.349				0.478			
Degree of differentiation	High/middle differentiation vs Low/undifferentiation	0.547				0.969			
Lymphovascular invasion	- vs +	0.030	11.239	1.258-100.394		0.046	3.547	1.023-12.295	
Never invasion	- vs +	0.452				0.969			
CEA(ng/ml)	0-5 vs >5	0.081	3.075	0.875-10.866		0.760			
CA199(U/ml)	0-37 vs>37	0.007	9.046	1.830-44.716		0.075			

KPS, Karnofsky Performance Status; GEJ, Gastroesophageal junction; AC, Adjuvant chemotherapy; NAC, Neoadjuvant chemotherapy.

To explore the relationship between NAC and the recurrence of HAS, we also conducted a univariate survival analysis of the DFS rates. The most common site of metastases was the liver. In the NAC-first group, 5 patients had postoperative liver metastasis with a median time of 5 months (1-58 months), 1 patient had lung metastasis at 1 month after surgery, and 2 patients metastasized to other sites. In the surgery-first group, postoperative liver metastasis occurred in 6 patients, with a median time of 7.5 months (4-26 months). In addition, 2 patients had lung metastasis at an average time of 10.5 months after surgery, 1 patient had ovarian metastasis at 18 months after gastrectomy, and 2 patients had metastases to other sites. The DFS time of the surgery-first group was substantially longer than that of the NAC-first group (**Figure 2A**, *P*=0.022). Specifically, the 1- and 3-year DFS rates of the NAC-first group were 80.4% and 71.5%, respectively. The 1- and 3-year DFS rates of the surgery-first group were 97% and 83.8%, respectively. Multivariate Cox regression analysis also revealed that clinical T4 (*P*=0.023) and lymphovascular invasion (*P*=0.046) was significant predictor of DFS outcomes (**Table 3**). However, a difference in the DFS rates between the NAC-first group and the surgery-first group was not found after PSM analysis (**Figure 2B**, *P*=0.108).

**Figure 3:**
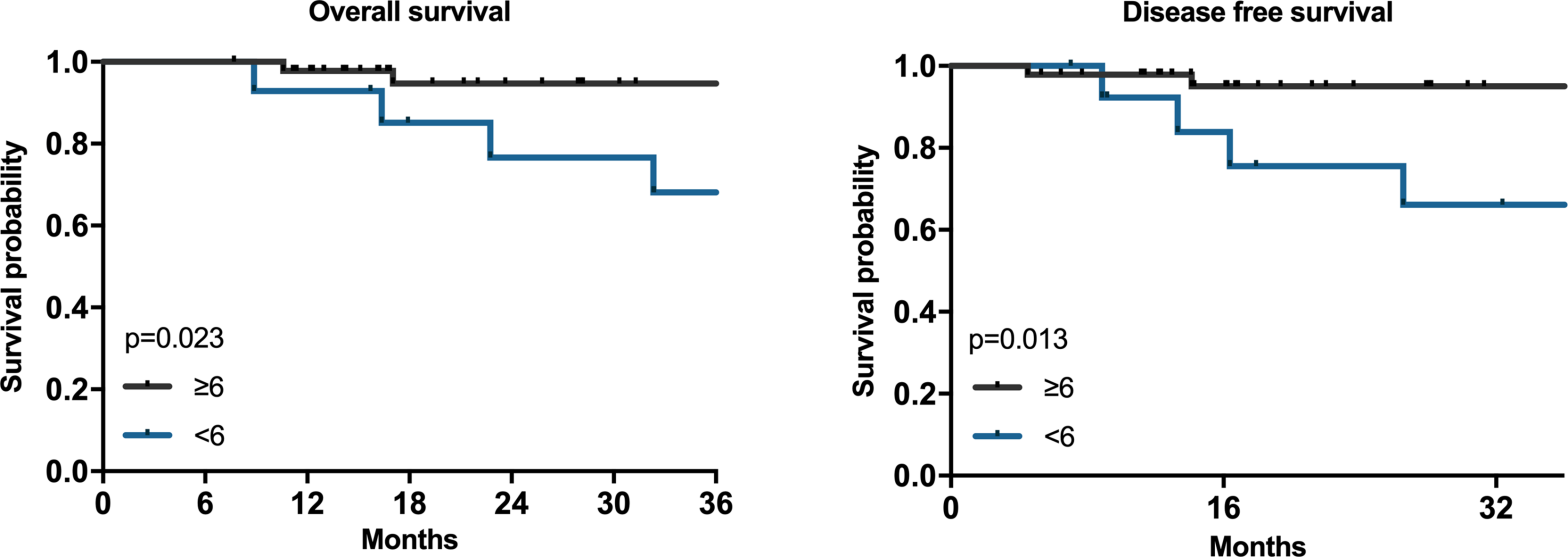
The associations between adjuvant chemotherapy circles and prognosis of HAS. Kaplan–Meier survival plots for adjuvant chemotherapy cycles ≥6 and<6 for 61 patients. P values were calculated by the log-rank test.

We also analyzed the relationship between the number of adjuvant chemotherapy cycles and the prognosis of HAS patients. Among the 61 patients who underwent surgery first, we found that the OS of the ≥6 cycles group were better than that of the <6 cycles group (**Figure 3**, *P*=0.023) and the DFS also had similar results (**Figure 3**, *P*=0.013).

### Prognostic Factors of HAS

As the clinical TNM stage was included in the survival analysis before and after PSM, there was confounding bias present. To reduce the confounding bias resulting from disease stage, we divided 100 patients into two groups, 29 patients in the NAC-first group and 71 patients in the surgery-first group. Their pathological TNM stages (pTNM, ypTNM) were used for univariate and multivariate survival analyses. In the NAC-first group, the results showed that the radiological response (P<0.01), the type of surgery (P = 0.032), and EGFR status (P=0.005) were related to the OS rate in the NAC-first group. Radiological response (P<0.01), number of LNs dissected (P = 0.039) and EGFR status (P=0.032) were related to the DFS rate (**Supplementary Table 1**). Multivariate Cox regression analysis showed that EGFR status was an independent risk factor for poor OS (P=0.006) and DFS outcomes (P=0.036) (**Supplementary Table 2**). In the surgery-first group, univariate survival analysis showed that age (p=0.03), lymphovascular invasion (P=0.045), CEA (P=0.044), and CA199 (P=0.003) were associated with the OS rate. Age (P=0.028), lymphovascular invasion (P=0.039), cycles of perioperative chemotherapy (P=0.029) and CA199 (P=0.001) were associated with the DFS rate (**Supplementary Table 3**). Multivariate Cox regression analysis showed that age (P=0.049) and CA199 (P=0.001) were independent risk factors for the DFS outcome (**Supplementary Table 4**).

## Discussion

Our research revealed that neoadjuvant chemotherapy (mainly platinum + fluorouracil) was not associated with increased survival of HAS patients undergoing radical surgery. However, our result was inconsistent with that of a previous study reported by Zeng et al, who declared that the DFS and disease-specific survival rates of patients in the NAC-first group were significantly higher than those in the surgery-first group (15). The conflicting results may be attributable to the different proportions of preoperative distant metastases. No patient was diagnosed with preoperative distant metastasis in our study, however, the proportion reached 70.5% in the Zeng et al. study (15). According to the result of our study, radical surgery was recommended for HAS patients without distant metastasis. However, the benefits of NAC with different regimens are still worthy of further research.

In our study, AC and lymphovascular invasion were two of the independent risk factors for DFS outcomes, which is similar to the conclusion of Zeng et al. (15). In a study by Qu, it was revealed that the survival time was not associated with sex, the disease location, or the serum AFP level (cutoff value: 40 ng/L), which is in agreement with our results (4). Similar to other studies of HAS, the results of Yang et al. indicated that pTNM is an independent risk factor for HAS (5, 24). In our study, the clinical or pathological stage was not an independent risk factor for prognosis. The statistical results might have been affected by the small sizes of the subgroups for pTNM stage, especially in the NAC-first group. The relatively short follow-up time may be another explanation. To understand the relationship between clinicopathological characteristics and the prognosis of HAS, it is still necessary to conduct multicenter studies with more samples to further study the treatment of HAS.

Our study demonstrated that AC was one of the independent factors for the prognosis of patients with HAS, similar to the findings of other studies (23, 31). However, few researchers have explored the optimal number of cycles of adjuvant chemotherapy that benefits patients with gastric cancer (32). As far as we know, the current research on HAS is blank. Due to the toxicity and side effects of neoadjuvant/adjuvant chemotherapy, it is necessary to determine the appropriate number of chemotherapy cycles to minimize side effects and maintain oncological efficacy, especially for patients with severe side effects. In our study, we found that patients who received ≥ 6 cycles of adjuvant chemotherapy had a better survival outcome than patients who received < 6 cycles, which is consistent with a multicenter retrospective study of gastric cancer (32). Accordingly, adjuvant chemotherapy is still advised, and more than 6 cycles of chemotherapy are preferable.

The incidence of Her-2 amplification in gastric cancer ranges from 6.0% to 29.5%; the variation may result from different testing methods and objective criteria (33). In our study, we found that the Her-2-positive expression rate of HAS was 22.89%, which is consistent with the results of previous studies that revealed a positivity rate of 25% (31). The negative prognostic value of Her-2 amplification for breast cancer is clear, however, opinions on its prognostic relationship with gastric cancer are still contradictory. In the ToGA study, researchers found that the OS and DFS of patients treated with trastuzumab combined with chemotherapy were better than those of patients treated with chemotherapy alone. Similarly, some researchers have suggested that trastuzumab combined with chemotherapy could improve OS outcomes (34). In our study, we also found that the two patients treated with chemotherapy and trastuzumab had the most satisfactory pathological response rate. Therefore, Her-2 inhibitors such as trastuzumab could be considered for NAC and the systematic treatment of HAS.

As the largest retrospective study on HAS treated with radical surgery, our study still had several limitations. Although 100 patients represent the largest sample size studied to date, this number was still small for statistical analysis. Furthermore, the chemotherapy regimens were various, especially for NAC, which may affect the results of NAC on HAS. Therefore, the conclusions drawn in our research should be adopted with caution.

## Conclusions

NAC based on platinum and fluorouracil may not improve the OS and DFS of patients with HAS treated with radical surgery. Patients who received more than 6 cycles of postoperative adjuvant chemotherapy had improved outcomes compared with the patient outcomes in other treatment groups. Therefore, the combination treatment of radical gastrectomy and sufficient adjuvant chemotherapy is recommended for patients with locally advanced HAS.

## Data Availability Statement

The original contributions presented in the study are included in the article/**Supplementary Material**. Further inquiries can be directed to the corresponding author.

## Ethics Statement

The studies involving human participants were reviewed and approved by Institutional Review Board of Peking University Cancer Hospital. The patients/participants provided their written informed consent to participate in this study. Written informed consent was obtained from the individual(s) for the publication of any potentially identifiable images or data included in this article.

## Author Contributions

KZ, methodology, data curation, investigation, formal analysis, writing - original draft, and writing - review and editing. AW, conceptualization, methodology, writing - review and editing, and funding acquisition. JW, conceptualization, data curation, investigation, and writing - review and editing. ZL, resources, data curation, and investigation. KJ, XJ, TF, ZJ, XW, and JZ, resources and investigation. ZB, conceptualization, resources, project administration, supervision, and writing - review and editing. All authors contributed to the article and approved the submitted version.

## Funding

This work was supported by the Peking University Clinical Scientist Program (BMU2019LCKXJ011), National Science Foundation for Young Scientists of China (No. 81802735), Beijing Youth Talent Plan (No. QML20191101), Science Foundation of Peking University Cancer Hospital (No. 2020-11) and Beijing Municipal Administration of Hospitals Clinical Medicine Development of Special Funding Support (XMLX202119).

## Conflict of Interest

The authors declare that the research was conducted in the absence of any commercial or financial relationships that could be construed as a potential conflict of interest.

## Publisher’s Note

All claims expressed in this article are solely those of the authors and do not necessarily represent those of their affiliated organizations, or those of the publisher, the editors and the reviewers. Any product that may be evaluated in this article, or claim that may be made by its manufacturer, is not guaranteed or endorsed by the publisher.
