# Supplementary figures and images for: The Value of Perioperative Chemotherapy for Patients With Hepatoid Adenocarcinoma of the Stomach Undergoing Radical Gastrectomy
Source: Front Oncol. 2022 Jan 10;11:789104. doi: 10.3389/fonc.2021.789104 (PMC8784750; doi:10.3389/fonc.2021.789104)

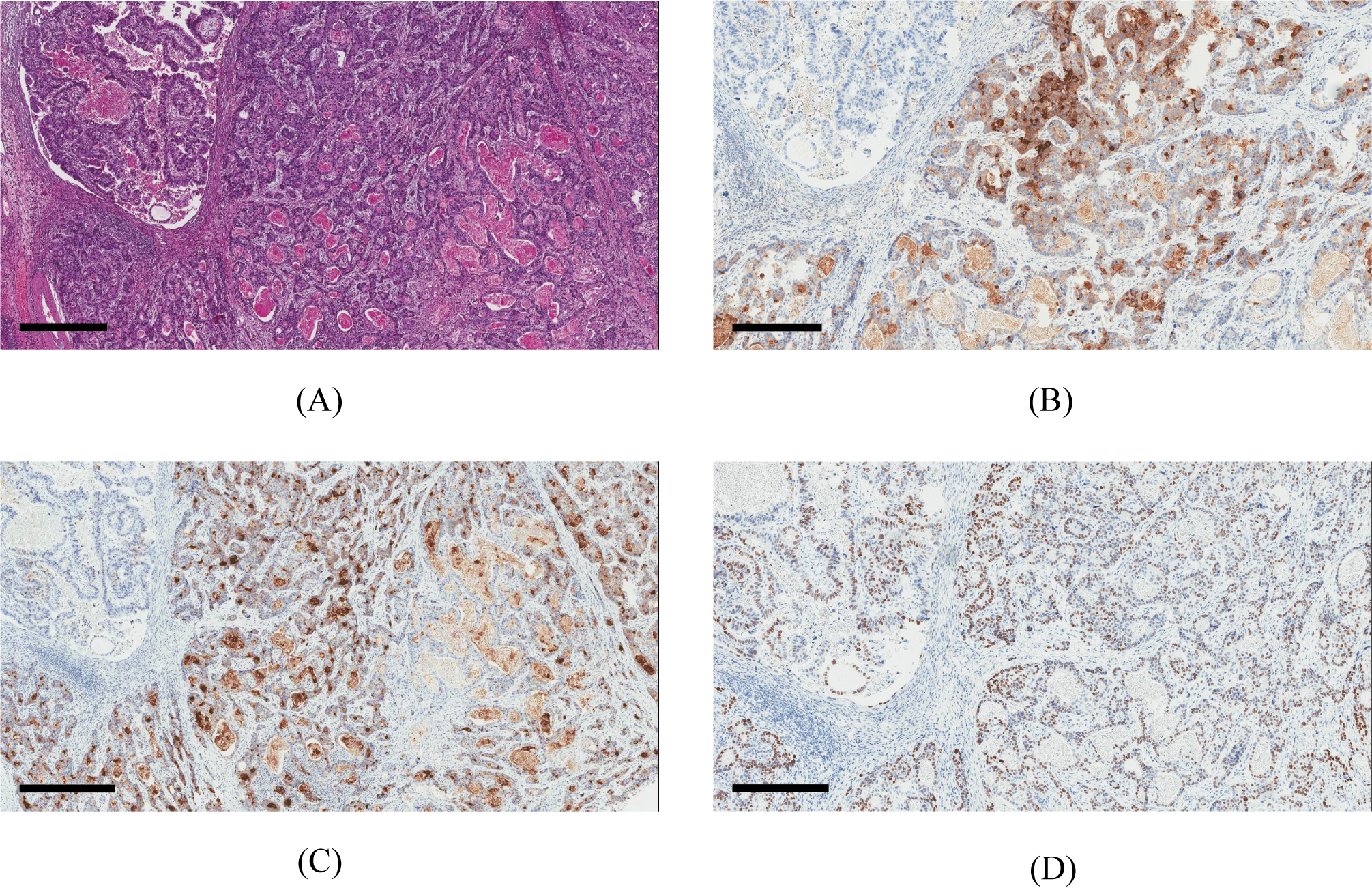

Supplement: Supplementary file 1 [file Image_1.tif]
