# Supplementary material for: The Value of Perioperative Chemotherapy for Patients With Hepatoid Adenocarcinoma of the Stomach Undergoing Radical Gastrectomy
Source: Front Oncol. 2022 Jan 10;11:789104. doi: 10.3389/fonc.2021.789104 (PMC8784750; doi:10.3389/fonc.2021.789104)
Supplement: Supplementary file 2 [file Table_1.docx]

Supplementary Material

# Surgery and adjuvant chemotherapy

All patients underwent laparotomy or laparoscopic gastrectomy with standard D2 lymph node dissection. Specific surgical gastrectomy was selected according to the location of the primary tumor, including subtotal gastrectomy and total gastrectomy, with 2 cases of total gastrectomy combined with resection of the pancreas tail plus splenectomy. The results of the postoperative pathological examination showed that the resection margins were all negative. Gastrointestinal reconstruction was performed according to the type of gastrectomy. Out of all of the cases, 86 patients received postoperative AC, among which 64 patients received platinum + fluorouracil, 4 patients received platinum + fluorouracil+ transcatheter arterial chemoembolization (TACE), 6 patients received platinum + fluorouracil +paclitaxel ± targeted chemotherapeutic drugs, 2 patients received paclitaxel + capecitabine (XP), 1 patient received DCF, 1 patient received etoposide + cisplatin (EP), 4 patients received Xeloda or tegafur capsules alone and 4 patients received other AC regimens. The other 10 patients refused AC, and AC regimen information was lacking for 4 patients. In the NAC-first group, 25 patients received AC, among whom 15 patients received platinum + fluorouracil, 6 patients received platinum + fluorouracil +paclitaxel ± targeted chemotherapeutic drugs, 1 patient received platinum + fluorouracil+ TACE, 1 patient received the XP regimen, 1 patient received the DCF regimen, and 1 patient received the EP regimen. The other 3 patients refused AC, and AC regimen information was lacking for 1 patient.

# A univariate survival analysis of 100 patients with HAS

We performed a univariate survival analysis of 100 patients with HAS. We found that age (*P*=0.031), NAC *(P*=0.02), number of dissected lymph nodes (*P*<0.001), number of perioperative chemotherapy cycles (*P*=0.02), surgery type (*P*<0.001) and CEA (*P*=0.021) were associated with OS (**Supplementary Table 5**). The OS time of the surgery-first group was better than that of the NAC-first group .The median OS for the entire study was 25.05 months (2.37-102.6 months). In the NAC-first group, the median OS time was 21.22 months (7.62-102.6 months). The median OS time for the surgery-first group was 28.19 months (2.37-97.48 months). The average survival time of the surgery-first group was 84.93 ±4.125 months and 61.90 ±11.629 months for the NAC-first group. We found that NAC (*P*=0.022), age (*P*=0.031), number of LNs dissected (*P*＜0.001), number of perioperative chemotherapy cycles (P=0.022) and CEA levels (P=0.031) were associated with DFS of HAS patients (**Supplementary Table 5**). The median DFS time for the entire study group was 22.03 months (2.37-102.6 months). In the NAC-first group, the median DFS time was 19.75 months (4.73-102.6 months). The median DFS time for the surgery-first group was 26.55 months (2.37-97.48 months). The average DFS time of the surgery-first group was 84.739 ±4.195 months, and the average DFS time of the NAC-first group was 61.589 ±12.280 months.

**3. Supplementary Figure**

**Supplementary Figure 1.** Pathological review of a typical hepatoid adenocarcinoma of the stomach sample.

**Legend:** Representative images of hematoxylin and eosin staining (A) and immunohistochemical staining of AFP (B), GPC3 (C) and SALL4 (D). AFP: alpha-fetoprotein, GPC3: glypican-3, SALL4: spalt like transcription factor 4. Scale bar: 500 μm (A,C), 300 μm (B,D).

**Supplementary Table 1**: Kaplan–Meier survival for NAC-first group of OS and DFS

| Clinicopathological features | | OS | DFS |
| --- | --- | --- | --- |
|  |  | P value | P value |
| Age(year) | 40-65 vs ＞65 | 0.2 | 0.307 |
| Gender | female | 0.824 | 0.881 |
|  | male |  |  |
| KPS score | 90 | 0.303 | 0.36 |
|  | 100 |  |  |
| Tumor location | GEJ | 0.221 | 0.325 |
|  | Non-GEJ |  |  |
| Family history of cancer | No | 0.74 | 0.567 |
|  | Yes |  |  |
| Radiological response | PR | 0 | 0 |
|  | SD |  |  |
|  | PD |  |  |
| TRG | 1 | 0.785 | 0.795 |
|  | 2 |  |  |
|  | 3 |  |  |
| ypT | 1 | 0.725 | 0.664 |
|  | 2 |  |  |
|  | 3 |  |  |
|  | 4a/b |  |  |
| ypN | 0 | 0.509 | 0.532 |
|  | 1-3 |  |  |
| ypTNM | I | 0.24 | 0.272 |
|  | II |  |  |
|  | III |  |  |
| Number of lymph node dissection | ＜16 | 0.068 | 0.039 |
|  | 16-30 |  |  |
|  | ＞30 |  |  |
| AC | No | 0.377 | 0.311 |
|  | Yes |  |  |
| cycles of perioperative chemotherapy | ＜6 | 0.13 | 0.093 |
|  | ≥6 |  |  |
| Borrmann type | I | 0.723 | 0.748 |
|  | II |  |  |
|  | III |  |  |
| Degree of differentiation | High / middle Differentiation | 0.935 | 0.926 |
|  | Low / undifferentiated |  |  |
| Lauren type | Intestinal type | 0.152 | 0.194 |
|  | Diffuse type |  |  |
|  | Mixed type |  |  |
| Surgery type | Proximal gastrectomy | 0.032 | 0.502 |
|  | Distal gastrectomy |  |  |
|  | Total gastrectomy |  |  |
| Vascular tumor thrombus | - | 0.334 | 0.236 |
|  | + |  |  |
| Never invasion | - | 0.525 | 0.646 |
|  | + |  |  |
| C-met | - | 0.079 | 0.121 |
|  | + |  |  |
|  | ++ |  |  |
| EGFR | - | 0.005 | 0.032 |
|  | + |  |  |
|  | ++ |  |  |
|  | +++ |  |  |
| HER-2 | -/± | 0.324 | 0.357 |
|  | ++ |  |  |
|  | +++ |  |  |
| MMR | pMMR | - | - |
| PDL1 | ≤5% | 0.487 | 0.359 |
|  | ＞5% |  |  |
| ki-67 | ＜25% | 0.275 | 0.121 |
|  | 25-49% |  |  |
|  | 50_75% |  |  |
|  | ＞75% |  |  |
| SALL4 | ＜25% | 0.805 | 0.784 |
|  | 25-49% |  |  |
|  | 50-75% |  |  |
|  | ＞75% |  |  |
| AFP | - | 0.245 | 0.51 |
|  | + |  |  |
| CEA (ng/ml) | 0-5 | 0.255 | 0.312 |
|  | ＞5 |  |  |
| CA199(U/ml) | 0-37 | 0.773 | 0.643 |
|  | ＞37 |  |  |
| CA242(U/ml) | 0-20 | 0.635 | 0.627 |
|  | ＞20 |  |  |
| CA72.4(U/ml) | 0-6.7 | 0.666 | 0.643 |
|  | ＞6.7 |  |  |
| CA125(U/ml) | 0-35 | - | - |
| AFP (ng/ml) | 0-7 | 0.726 | 0.672 |
|  | ＞7 |  |  |

NAC: neoadjuvant chemotherapy, AC: adjuvant chemotherapy,

KPS: Karnofsky Performance Status, TRG: tumor regression grade,

ypT: pathological T stage after neoadjuvant chemotherapy

ypN: pathological N stage after neoadjuvant chemotherapy

ypTNM: pathological TNM stage after neoadjuvant chemotherapy

MMR: mismatch repair genes, PDL-1: Programmed cell death 1 ligand 1

**Supplementary Table 2**: Multivariate analysis of OS and DFS of NAC-first group

| Clinicopathological features | | OS | DFS |
| --- | --- | --- | --- |
|  |  | P value | Pvalue |
| Radiological response | PR | 1.00 | 1.00 |
|  | SD |  |  |
|  | PD |  |  |
| TRG degree | 1 | 0.79 | 0.783 |
|  | 2 | 0.728 | 0.824 |
|  | 3 | 0.959 | 0.847 |
| ypT | 1 | 0.699 | 0.438 |
|  | 2 | 0.326 | 0.136 |
|  | 3 | 0.956 | 0.81 |
|  | 4 | 0.586 | 0.589 |
| ypN | 0 vs 1-3 | 0.994 | 0.889 |
| ypTNM | I | 0.713 | 0.83 |
|  | II | 0.669 | 0.749 |
|  | III | 0.945 | 0.969 |
| AC |  | 0.685 | 0.552 |
| Cycles of perioperative chemotherapy | ＜6 vs ≥6 | 0.184 | 0.133 |
| Surgery type | Proximal gastrectomy | 0.051 | - |
|  | Distal gastrectomy | 0.282 | - |
|  | Total gastrectomy | 0.764 | - |
| Vascular tumor thrombus | - vs + | 0.265 | 0.2 |
| Never invasion | - vs + | 0.52 | 0.618 |
| EGFR | - | 0.006 | 0.036 |
|  | + | 0.145 | 0.14 |
|  | ++ | 0.888 | 0.864 |
|  | +++ | 0.106 | 0.103 |
| Number of lymph node dissection | ＜16 | 0.066 | 0.328 |
|  | 16-30 | 0.963 | 0.936 |
|  | ＞30 | 0.414 | 0.423 |
| Degree of differentiation | High/middle differentiation vs Low / undifferentiation | 0.567 | 0.356 |
| C-met | - | 0.062 | - |
|  | + | 0.018 | - |
|  | ++ | 0.05 | - |

NAC: neoadjuvant chemotherapy, AC: adjuvant chemotherapy, TRG: tumor

regression grade

ypT: pathological T stage after neoadjuvant chemotherapy

ypN: pathological N stage after neoadjuvant chemotherapy

ypTNM: pathological TNM stage after neoadjuvant chemotherapy

**Supplementary Table 3**: Kaplan–Meier survival for surgery-first group of OS and DFS

| Clinicopathological features | | OS | DFS |
| --- | --- | --- | --- |
|  |  | P value | P value |
| Age(year) | ≤40 | 0.03 | 0.028 |
|  | 40-65 |  |  |
|  | ＞65 |  |  |
| Gender | female | 0.329 | 0.25 |
|  | male |  |  |
| KPS score | 80 | 0.932 | 0.909 |
|  | 90 |  |  |
|  | 100 |  |  |
| Tumor location | GEJ | 0.691 | 0.732 |
|  | Non-GEJ |  |  |
| Family history of cancer | No | 0.769 | 0.86 |
|  | Yes |  |  |
| pT | 1 | 0.501 | 0.473 |
|  | 2 |  |  |
|  | 3 |  |  |
|  | 4a/b |  |  |
| pN | 0 | 0.353 | 0.359 |
|  | 1-3 |  |  |
| pTNM | I | 0.63 | 0.609 |
|  | II |  |  |
|  | III |  |  |
| AC | No | 0.516 | 0.51 |
|  | Yes |  |  |
| Number of lymph node dissection | 16-30 | 0.105 | 0.105 |
|  | ＞30 |  |  |
| Cycles of perioperative chemotherapy | 0 | 0.051 | 0.029 |
|  | ＜6 |  |  |
|  | ≥6 |  |  |
| Borrmann type | I | 0.271 | 0.326 |
|  | II |  |  |
|  | III |  |  |
|  | IV |  |  |
| Degree of differentiation | High / middle differentiation | 0.344 | 0.311 |
|  | Low / undifferentiation |  |  |
| Lauren type | Intestinal type | 0.735 | 0.738 |
|  | Diffuse type |  |  |
|  | Mixed type |  |  |
| Surgery type | Proximal gastrectomy | - | 0.596 |
|  | Distal gastrectomy |  |  |
|  | Total gastrectomy |  |  |
| Lymphatic and vascular tumor thrombus | - | 0.045 | 0.039 |
|  | + |  |  |
| Never invasion | - | 0.347 | 0.337 |
|  | + |  |  |
| C-met | - | 0.662 | 0.598 |
|  | + |  |  |
|  | ++ |  |  |
|  | +++ |  |  |
| EGFR | - | 0.932 | 0.923 |
|  | + |  |  |
|  | ++ |  |  |
|  | +++ |  |  |
| Her-2 | -/± | 0.434 | 0.478 |
|  | ++ |  |  |
|  | +++ |  |  |
| MMR | pMMR | 0.782 | 0.798 |
|  | dMMR |  |  |
| PDL1 | ≤5% | 0.596 | 0.578 |
|  | ＞5% |  |  |
| ki-67 | ＜25% | 0.172 | 0.252 |
|  | 25-49% |  |  |
|  | 50_75% |  |  |
|  | ＞75% |  |  |
| SALL4 | ＜25% | 0.815 | 0.815 |
|  | 25-49% |  |  |
|  | 50-75% |  |  |
|  | ＞75% |  |  |
| AFP | - | 0.774 | 0.8 |
|  | + |  |  |
| CEA(ng/ml) | 0-5 | 0.044 | 0.063 |
|  | ＞5 |  |  |
| CA199(U/ml) | 0-37 | 0.003 | 0.001 |
|  | ＞37 |  |  |
| CA242(U/ml) | 0-20 | 0.251 | 0.243 |
|  | ＞20 |  |  |
| CA72.4(U/ml) | 0-6.7 | 0.907 | 0.928 |
|  | ＞6.7 |  |  |
| CA125(U/ml) | 0-35 | - | - |
| AFP((ng/ml) | 0-7 | 0.279 | 0.226 |
|  | ＞7 |  |  |

AC: adjuvant chemotherapy, KPS: Karnofsky Performance Status, pT: pathological T Stage, pN: pathological N stage, pTNM: pathological TNM stage, MMR: mismatch repair genes, PDL-1: Programmed cell death 1 ligand 1

**Supplementary Table 4**: Multivariate analysis of OS and DFS of surgery-first group

| Clinicopathological features | | OS | DFS |
| --- | --- | --- | --- |
|  |  | P value | P value |
| Age(year) | ≤40 | 0.207 | 0.049 |
|  | 40-65 | 0.17 | 0.055 |
|  | ＞65 | 0.08 | 0.015 |
| pT | 1 | 0.719 | 0.628 |
|  | 2 | 0.38 | 0.326 |
|  | 3 | 0.376 | 0.28 |
|  | 4a/b | 0.989 | 0.907 |
| pN | 0 vs 1-3 | 0.733 | 0.739 |
| pTNM | I | 0.486 | 0.658 |
|  | II | 0.245 | 0.399 |
|  | III | 0.393 | 0.555 |
| Lymphatic and vascular tumor thrombus | - vs + | 0.973 | 0.973 |
| Never invasion | - vs + | 0.878 | 0.852 |
| CEA(ng/ml) | 0-5 vs ＞5 | 0.291 | 0.502 |
| CA199(U/ml) | 0-37 vs ＞37 | 0.002 | 0.001 |
| AC | No vs Yes | 0.631 | 0.615 |
| cycles of perioperative chemotherapy | 0 | - | 0.510 |
|  | ＜6 | - | 0.276 |
|  | ≥6 | - | 0.454 |

AC: adjuvant chemotherapy, pT: pathological T stage, pN: pathological N stage, pTNM: pathological TNM stage

**Supplementary Table 5**: Univariate survival analysis of OS and DFS before PSM

| Clinicopathological features |  | Before PSM of 100 patients | |
| --- | --- | --- | --- |
|  |  | OS | DFS |
|  |  | *P* value | *P* value |
| Age(year) | ≤40  40-65  ＞65 | 0.031 | 0.031 |
| Gender | female | 0.287 | 0.208 |
|  | male |  |  |
| KPS score | 80 | 0.872 | 0.864 |
|  | 90 |  |  |
|  | 100 |  |  |
| Tumor location | GEJ | 0.071 | 0.102 |
|  | Non-GEJ |  |  |
| Family history of cancer | No | 0.864 | 0.75 |
|  | Yes |  |  |
| NAC | NO | 0.02 | 0.022 |
|  | Yes |  |  |
| cT | 1 | 0.3 | 0.34 |
|  | 2 |  |  |
|  | 3 |  |  |
|  | 4a/b |  |  |
| cN | 0 | 0.647 | 0.526 |
|  | 1-3 |  |  |
| Clinical TNM stage | IIa | 0.768 | 0.735 |
|  | IIb |  |  |
|  | III |  |  |
|  | IVa |  |  |
| Number of lymph node dissection | ＜16 | 0 | 0 |
|  | 16-30 |  |  |
|  | ＞30 |  |  |
| AC | No | 0.39 | 0.382 |
|  | Yes |  |  |
| cycles of perioperative chemotherapy | 0 | 0.020 | 0.022 |
|  | ＜6 |  |  |
|  | ≥6 |  |  |
| Borrmann type | I | 0.21 | 0.283 |
|  | II |  |  |
|  | III |  |  |
|  | IV |  |  |
| Degree of differentiation | High / middle differentiation | 0.612 | 0.545 |
|  | Low / undifferentiation |  |  |
| Lauren type | Intestinal type | 0.887 | 0.865 |
|  | Diffuse type |  |  |
|  | Mixed type |  |  |
| Surgery type | Proximal gastrectomy | 0 | 0.065 |
|  | Distal gastrectomy |  |  |
|  | Total gastrectomy |  |  |
| Lymphatic and vascular tumor thrombus | - | 0.12 | 0.094 |
|  | + |  |  |
| Never invasion | - | 0.472 | 0.493 |
|  | + |  |  |
| C-met | - | 0.38 | 0.379 |
|  | + |  |  |
|  | ++ |  |  |
|  | +++ |  |  |
| EGFR | - | 0.709 | 0.727 |
|  | + |  |  |
|  | ++ |  |  |
|  | +++ |  |  |
| HER-2 | -/± | 0.652 | 0.514 |
|  | ++ |  |  |
|  | +++ |  |  |
| MMR | pMMR | 0.732 | 0.744 |
|  | dMMR |  |  |
| PDL1 | ≤5% | 0.851 | 0.829 |
|  | ＞5% |  |  |
| ki-67 | ＜25% | 0.089 | 0.102 |
|  | 25-49% |  |  |
|  | 50_75% |  |  |
|  | ＞75% |  |  |
| SALL4 | ＜25% | 0.626 | 0.631 |
|  | 25-49% |  |  |
| AFP | 50-75% |  |  |
|  | ＞75% |  |  |
|  | - | 0.601 | 0.554 |
|  | + |  |  |
| CEA(ng/ml) | 0-5 | 0.021 | 0.031 |
|  | ＞5 |  |  |
| CA199(U/ml) | 0-37 | 0.065 | 0.053 |
|  | ＞37 |  |  |
| CA242(U/ml) | 0-20 | 0.465 | 0.47 |
|  | ＞20 |  |  |
| CA72.4(U/ml) | 0-6.7 | 0.656 | 0.609 |
|  | ＞6.7 |  |  |
| CA125 (U/ml)  AFP(ng/ml) | 0-35 |  |  |
|  | 0-7 | 0.513 | 0.459 |
|  | ＞7 |  |  |

KPS: Karnofsky Performance Status, GEJ: Gastroesophageal junction, AC: adjuvant chemotherapy, NAC: neoadjuvant chemotherapy
